# The Effects of Alkyl Chain Combinations on the Structural and Mechanical Properties of Biomimetic Ion Pair Amphiphile Bilayers

**DOI:** 10.3390/bioengineering4040084

**Published:** 2017-10-11

**Authors:** Cheng-hao Chen, Ching-an Tian, Chi-cheng Chiu

**Affiliations:** Department of Chemical Engineering, National Cheng Kung University, Tainan City 701, Taiwan; a124039457@gmail.com (C.C.); happy122025@gmail.com (C.T.)

**Keywords:** biomimetic bilayer, ion pair amphiphile, molecular dynamics

## Abstract

Ion pair amphiphile (IPA), a lipid-like complex composed of a pair of cationic and anionic surfactants, has great potentials in various pharmaceutical applications. In this work, we utilized molecular dynamics (MD) simulation to systematically examine the structural and mechanical properties of the biomimetic bilayers consist of alkyltrimethyl-ammonium-alkylsulfate (C_m_TMA^+^-C_n_S^−^) IPAs with various alkyl chain combinations. Our simulations show an intrinsic one-atom offset for the C_m_TMA^+^ and C_n_S^−^ alignment, leading to the asymmetric index definition of ΔC = m − (n + 1). Larger |ΔC| gives rise to higher conformational fluctuations of the alkyl chains with the reduced packing order and mechanical strength. In contrast, increasing the IPA chain length enhances the van der Waals interactions within the bilayer and thus improves the bilayer packing order and mechanical properties. Further elongating the C_m_TMA^+^-C_n_S^−^ alkyl chains to m and n ≥ 12 causes the liquid disorder to gel phase transition of the bilayer at 298 K, with the threshold membrane properties of 0.45 nm^2^ molecular area, deuterium order parameter value of 0.31, and effective bending rigidity of 20 k_B_T, etc. The combined results provide molecular insights into the design of biomimetic IPA bilayers with wide structural and mechanical characteristics for various applications.

## 1. Introduction

**Figure 1:**
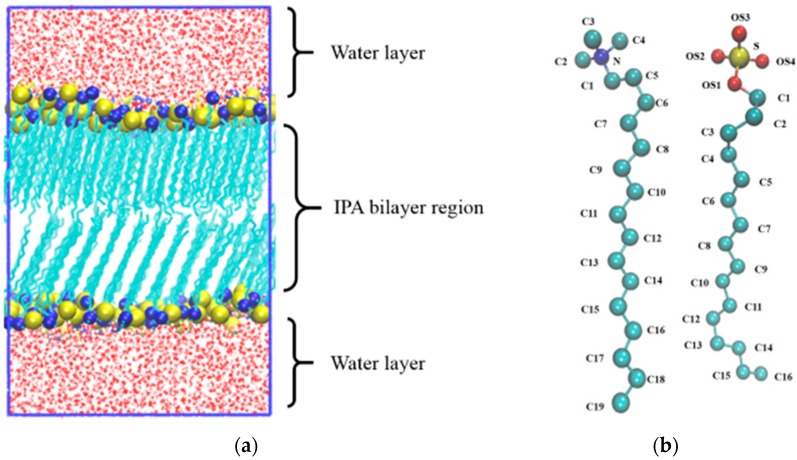
(**a**) A representative initial configuration of the biomimetic ion pair amphiphile bilayer systems for molecular dynamics (MD) simulation. The presented ion pair amphiphile (IPA) bilayer is composed of hexadecyltrimethyl-ammonium-dodecylsulfate (HTMA-HS) IPA, i.e., C_16_TMA^+^-C_16_S^−^. (**b**) The molecular structure of HTMA-HS IPA. Atomic color codes are: nitrogen in blue, sulfur in yellow, oxygen in red, and carbon in turquoise, respectively. Hydrogen atoms are not shown for clarity. Graphics were made using VMD [24].

Phospholipids are the major amphiphilic components of the cell membrane and can self-assemble in vitro into vesicles, hollow spherical structures formed by wrapping bilayers [1]. Lipid vesicles, also termed liposomes, are considered as promising drug-delivery vehicles that can carry either hydrophobic drugs in the alkyl chain region or aqueous drugs in its inner cavities filled with aqueous solution [1,2]. However, the high production cost of liposomes has limited their practical uses [2,3]. Recently, vesicles constituted of cationic and anionic surfactants, termed catanionic vesicles, have been proposed as low cost alternatives for liposomes [4]. Yet, the counter ions released from the surfactant mixture compensate their biocompatibility [5]. Several approaches have been developed to remove the counterions, such as precipitation, proton exchange, and ion-exchange methods [6,7,8,9]. Removing the counter ions in the catanionic mixture results in a novel amphipathic complex termed an ion pair amphiphile (IPA), which consists of a pair of cationic and anionic surfactants, as illustrated in Figure 1b. Compared with phospholipids with two covalently bonded hydrocarbon chains, an IPA complex is considered as a pseudo double-chained amphiphile where the two alkyl chains are held together via non-bonded electrostatic interaction between charged hydrophilic groups. The structural similarities between an IPA and phospholipid can translate into common self-assembled structures, such vesicles and lamellar structures [8]. Due to the easiness of manufacturing and composition variations, IPA complexes have great potentials in various applications including cosmetics, drug delivery, and DNA transfection, etc. [7,10,11]. 

An IPA bilayer shows similar thermotropic phase transition behaviors as a lipid bilayer with two main phases: a solid-like gel (S) phase in which alkyl chains are orderly oriented, and a fluidic liquid-disordered (Ld) phase in which alkyl chains are loosely packed [12]. The phase transition of a bilayer occurs at the main transition temperature, T_m_, and a bilayer can transition from S to Ld phase beyond T_m_. Compared to a bilayer in tightly packed S state, a bilayer in Ld state has more disordered alkyl chain conformations and smaller mechanical properties [12,13,14,15,16,17]. Recently, Lee and coworkers prepared several IPA complexes of the alkyltrimethylamine–alkylsulfate with different alkyl chain combinations and studied the thermotropic phase transition behavior of the corresponding IPA bilayers [12]. They found that the bilayer T_m_ value increases with the total alkyl number of IPA. This is consistent with that observed for the phosphatidylcholines (PC) bilayer systems. In addition, IPA bilayers exhibit much higher transition temperature compared with the corresponding PC systems.

In addition to the experimental approaches, many studies have applied computer simulations to examine the structures and the phase behaviors of both the phospholipid and IPA bilayer systems [18,19,20,21,22]. Tu et al. used all-atom molecular dynamics (MD) simulation to characterize the structure of the DPPC (1,2-dipalmitoyl-sn-glycero-3-phosphocholine) bilayer and successfully reproduced the experimental observations including area per lipid, lamellar spacing, alkyl chain tilt angle, and gauche fraction of dihedral segments along alkyl chains [18]. Their MD results further provided the detailed conformations of the head groups and the carbon segments that were not easily accessible experimentally. Kindt et al. utilized MD to investigate the effects of lipid glycerol backbone packing on the alkyl tilt angles for S phase lipid bilayers. Their results indicated that a S phase lipid bilayer exhibits randomized backbone orientations [19]. More recently, Kuo et al. conducted a series of all-atom MD simulations to investigate the structure of IPA bilayers composed of hexadecyl-trimethylammonium-dodecylsulfate (HTMA-DS) IPA and a double-tailed cationic amphiphile, ditetradecyl-dimethylammonium chloride (DTDAC) [20]. They showed that introducing DTDAC complements the alkyl chain asymmetry between HTMA and DS to achieve an ordered structure of mixed HTMA-DS/DTDAC bilayers. Later, Kuo and Chang also investigate the bilayer structural properties composed of hexadecyltrimethylammonium-dodecylsulfate (HTMA-DS) IPA and cholesterol [21]. The addition of cholesterol leads to the conformational disorder of IPA components where HTMA^+^ protruded above the membrane surface, and DS^−^ shifted toward the inside of IPA bilayer. In addition, the void near the hydrophobic core of the HTMA-DS bilayer induced by cholesterol increases the conformational freedom of the alkyl terminal segments. Extended from Kuo’s work, a MD study by Huang et al. revealed that the biased interaction between cholesterol and anionic alkylsulfate leads to diverse structural and mechanical effects of cholesterol on the HTMA-DS and DTMA-HS bilayers [22]. These MD studies provide detailed insights into structural properties of IPA/lipid bilayers at the molecular level to further complement of the experimental observations.

The thermotropic T_m_ data reported by Lee et al. illustrated that the IPA systems with the same total number of carbon atoms have different transition temperature [12]. For example, the reported T_m_ for dodecyltrimethylammonium-tetradecylsulfate (DTMA-TS) bilayer is 330.05 K, and that for tetradecyltrimethylammonium-dodecylsulfate (TTMA-DS) bilayer is 322.80 K [12]. These results gave rise to an interesting question: what are the effects of alkyl chain asymmetry and total alkyl chain length on the IPA bilayer properties other than T_m_ differences? In order to elucidate the combined effects of IPA alkyl chain asymmetry and total chain length, we applied MD simulations to study the bilayer systems composed of alkyltrimethylammonium-alkylsulfate IPA series, i.e., C_m_TMA^+^-C_n_S^−^. With various m and n combinations where m and n ranged from 10 to 16, we examined the effects of asymmetric IPA composition and the total alkyl chain length on the IPA bilayer structural and mechanical properties.

## 2. Materials and Methods

The chosen IPAs to compose the bilayer systems were alkyltrimethyl-ammonium-alkylsulfate, i.e., C_m_TMA^+^-C_n_S^−^. To complement with experimental data by Lee et al. [12], we selected the (m, n) combinations based on the experimental test sets but more thoroughly in the range of 10 to 16, as listed in Table 1. The initial configuration for an IPA bilayer system were constructed using PACKMOL software [23]. For each IPA complex of C_m_TMA^+^-C_n_S^−^ the nitrogen and sulfur atoms of the cationic and anionic amphiphiles, respectively, were placed at a close distance of approximately 4.3 Å as described in the previous work [21]. Each bilayer system was composed of 128 IPA surfactants and 3464 water molecules with the bilayer normal aligned with the z-axis. Figure 1 shows the representative configuration of the HTMA-HS (C_16_TMA^+^-C_16_S^−^) bilayer system. 

Each IPA system was first energy minimized via the steepest descent minimization algorithm, then equilibrated at 348 K and 1 bar for 30 ns at which the IPA bilayer was in liquid disordered (Ld) phase. The IPA bilayer system was annealed from 333 K to 298 K with a 2.5 K/ns cooling rate, allowing the bilayer to naturally transition to gel phase, if the phase transition occurred. After the annealing process, the bilayer system was equilibrated at 298 K and 1 bar for 200 ns, which is typical for MD simulations of bilayers [18,19,20,21,22]. The system coordinates were saved every 10 ps during the last 50 ns period for further analyses of the bilayer structural and mechanical characteristics. The system equilibrations were verified via the convergence of various membrane properties as demonstrated in Figures S1 and S2 of Supplementary Materials.

All molecular dynamics (MD) simulations were carried out using Gromacs 5.0.4 simulation package [25,26]. Periodic boundary conditions were applied in all three dimensions. The CHARMM36 united-atom (C36-UA) force field parameters were applied for IPA molecules and the TIP3P model for water [27,28,29]. Simulations with C36-UA force field have been shown accurately reproduced lipid bilayer and micelle structures compared with experimental data and C36 MD results with less computation time [28]. Representative molecular topologies for C_16_TMA^+^ and C_16_S^−^ are given in Tables S1 and S2 of Supplementary Materials. All simulations were performed under isothermal-isobaric (NPT) ensemble. The temperature and pressure of the simulation systems were maintained at 298 K and 1 bar via Nosé-Hoover thermostat and semi-isotropic Parrinello-Rahman barostat, respectively [30,31,32]. The Lennard-Jones pair potentials were smoothly switched to 0 starting from 0.8 nm and cut off at 1.2 nm. The long-range electrostatic potential was evaluated using the Particle-mesh Ewald (PME) method [33]. Each bond was constrained at its equilibrium bond length using the linear constraint solver (LINCS) algorithm [34]. An integration time step of 2 fs was applied to evaluate the equations of motion for atoms.

## 3. Results and Discussion

### 3.1. Phase Behaviors of the C_m_TMA^+^-C_n_S^−^ IPA Bilayers at 298 K

**Figure 2:**
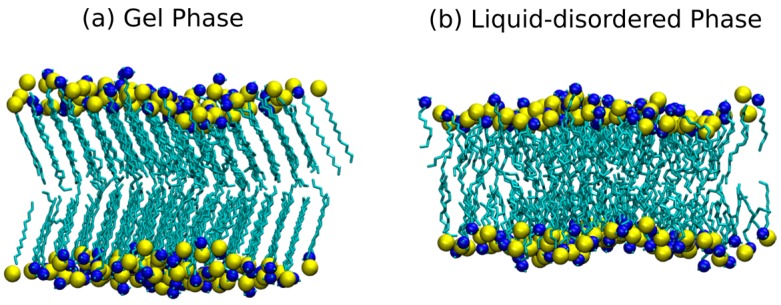
The representative snapshots of an IPA bilayer (**a**) in gel (S) phase or (**b**) in liquid-disordered (Ld) phase. The head group atoms, nitrogen and sulfur, are drawn in blue and yellow, respectively; the alkyl chains are colored in cyan. Graphics were made using visual molecular dynamics (VMD) [24].

As described in the Method Section, all the IPA systems were first equilibrated at 348 K to ensure all bilayers systems were in the liquid-disordered (Ld) phase. Followed by the annealing process to gradually cool down to 298 K, the IPA bilayer could transit into the solid-like gel (S) phase or remain in Ld phase, depending on the molecular nature of IPA. As illustrated in Figure 2, an IPA bilayer shows orderly alkyl chain orientations in the gel phase; whereas in the Ld phase, IPA molecules have more freedom of motion and are loosely packed, leading to a more disordered bilayer structure. The phase behaviors of the C_m_TMA^+^-C_n_S^−^ IPA bilayers at 298 K can therefore be distinguished via visualizing the simulation trajectories. Our results indicate that at 298 K the bilayers composed with C_m_TMA^+^-C_n_S^−^ IPA with m or n ≤ 10 are in the fluidic Ld phase, otherwise in the S phase, as shown by the boundary line in Table 2. Combined with the analyses described in the following sections, we characterized the effects of various IPA alkyl chain combinations on the structural and mechanical properties of IPA bilayers in different phases, as summarized in Table 2. 

### 3.2. Alkyl Chain Asymmetry Definition

**Figure 3:**
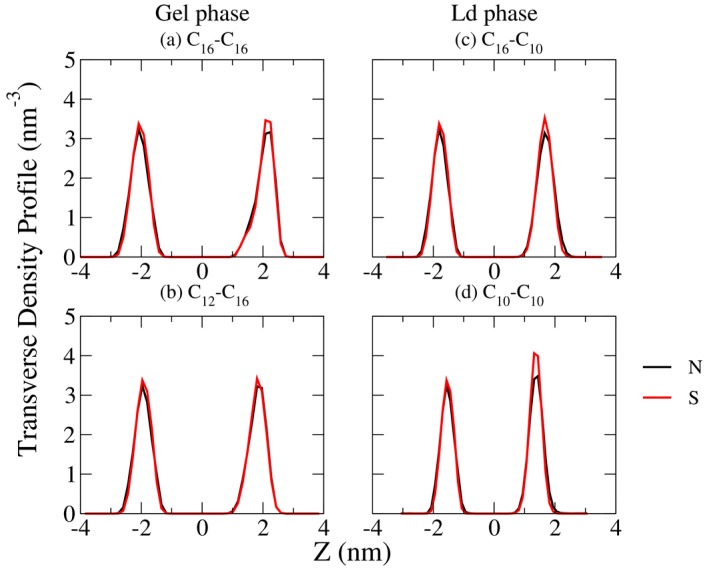
Representative transverse density profiles of nitrogen and sulfur of the charged head groups for the IPA systems of (**a**) C_16_TMA^+^-C_16_S^−^ and (**b**) C_12_TMA^+^-C_16_S^−^ in gel (S) phase, and (**c**) C_16_TMA^+^-C_10_S^−^ and (**d**) C_10_TMA^+^-C_10_S^−^ systems in liquid-disordered (Ld) phase.

Conventionally, the C_m_TMA^+^-C_n_S^−^ IPAs with m = n are considered as symmetric such as C_16_TMA^+^-C_16_S^−^ (HTMA-HS), and m ≠ n as asymmetric such as C_16_TMA^+^-C_12_S^−^ (HTMA-DS). To inspect the atomic alignment between the two components with in an IPA complex, we analyzed the transverse density profile for each IPA bilayer system, as shown in Figure 3 and Figures S3–S5 of Supplementary Materials. The resulting profiles clearly reveal that, regardless of the m and n combinations or the phase state of the bilayer, the nitrogen and sulfur atoms of the charged head groups are aligned on the same height. Thus, the first alkyl carbon atom of the cationic surfactant aligns with the sulfate oxygen atom of the anionic surfactant bridging the sulfate group and alkyl chain. This leads to one carbon atom mismatch between the two alkyl chains. Therefore, we defined the alkyl chain asymmetric index ΔC as follows:

ΔC = m − (n + 1),(1)

to quantify the degree of IPA asymmetry for the C_m_TMA^+^-C_n_S^−^ IPA series. Higher |ΔC| value corresponds to greater alkyl chain asymmetry. As listed in Table 1, all the tested C_m_TMA^+^-C_n_S^−^ IPA systems, including the ones with m = n combination, are asymmetric due to the intrinsic molecular structures of the composing surfactants.

### 3.3. IPA Bilayer Structural Properties

**Figure 4:**
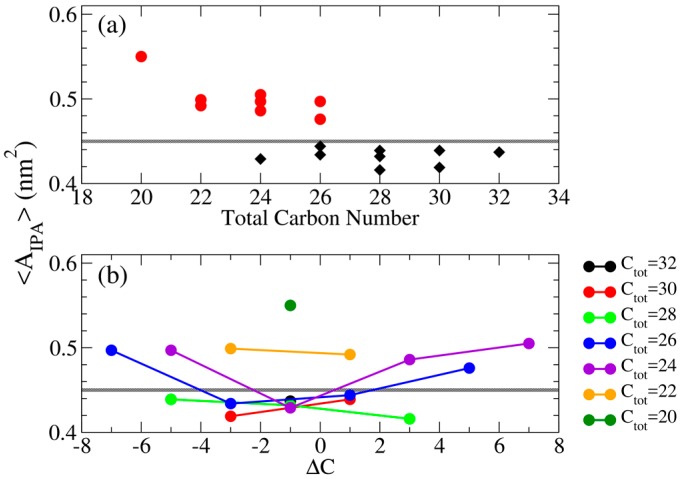
Averaged molecular area <A_IPA_> as function of (**a**) total alkyl carbon number (top panel) in which bilayers in S and Ld phases are colored in black diamonds and red dots, respectively, and as function of (**b**) ΔC (bottom panel) in which each line represents systems with equal total alkyl carbon numbers. The gray lines represent the threshold value of A_IPA_ = 0.45 nm^2^ to distinguish the Ld and S phases.

In our MD simulation, the cross-sectional area of the simulation box, i.e., the *x*-*y* area, was equivalent to the lateral area of the IPA bilayer. Hence, the equilibrium area per IPA complex, A_IPA_ can be calculated as follows:

A_IPA_ = <A>/N (2)

where <A> is the average lateral bilayer area calculated from the last 50 ns simulation, and N = 64 denotes the number of IPA molecule in a single leaflet. As summarized in Table 1, IPA bilayers in the S phase have smaller molecular area indicating a tight packing; whereas the one in the Ld phase has larger molecular area reflecting a more loosely structural packing. The molecular area for the S phase IPA systems varies from 0.415 to 0.45 nm^2^, and the one for the Ld Phase IPA bilayer has the value range of 0.47~0.55 nm^2^ which is much larger than the former. Therefore, the molecular area value of 0.45 nm^2^ can be used to distinguish the gel state and the Ld phase IPA bilayers, as the boundary line depicted in Table 2. As illustrated in Figure 4, A_IPA_ decreases with increased total alkyl carbon number, mainly due to the increased v.d.W interaction between longer alkyl chains. Furthermore, increasing |ΔC| can lead to increased A_IPA_, suggesting a less dense molecular packing within the bilayer induced by the alkyl chain mismatch.

**Figure 5:**
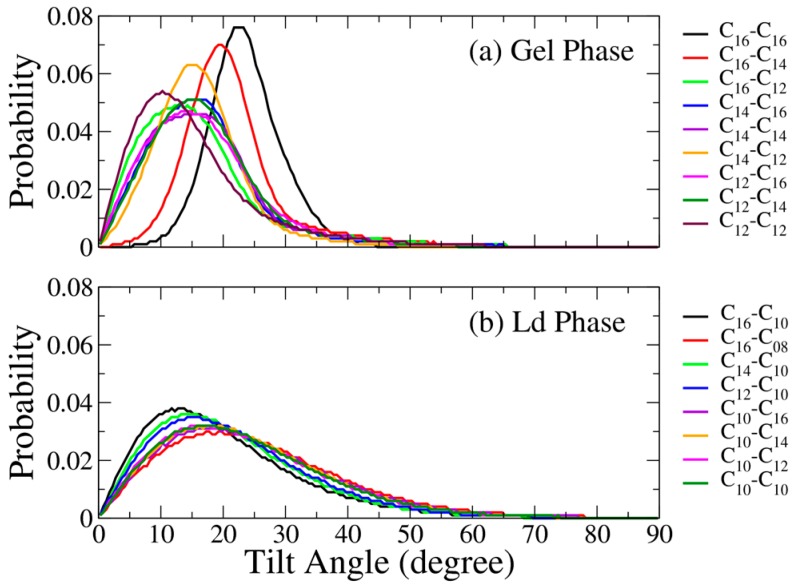
Tilt angle distribution for all the tested C_m_TMA^+^-C_n_S^−^ IPA bilayer systems (**a**) in gel (S) phase and (**b**) in liquid-disordered (Ld) phase.

The orientations of the alkyl chain within the hydrophobic region of a bilayer can be characterized using the alkyl chain tilt angles, which can be evaluated as the angle between the directive vector of the alkyl chain and the bilayer normal. Here, the directive vector was defined as the vector connecting the first to the second last carbon atoms of the alkyl chain for either cationic or anionic surfactant. Figure 5 displays the tilt angle distributions for all the tested C_m_TMA^+^-C_n_S^−^ IPA bilayers. The IPA systems in the Ld phase show broader tilt angle distributions, whereas the ones in the S phase have narrower tilt angle distributions. Such variation arises from the packing order difference between the two phases. The IPA molecules in the Ld phase bilayer are packed loosely resulting in greater freedoms for alkyl chain tilting. Note that the tilt angle distributions of all the IPA systems in the Ld phase are similar, as shown in Figure 5b. However, in the S phase, IPA systems show different tilt angle distributions. Higher amount of total carbon numbers generally leads to greater tilt angle for IPA bilayers in gel phase. Furthermore, the IPA bilayers composed with IPAs of greater |ΔC| value, i.e., greater alkyl chain mismatch, exhibit smaller tilt angles. This is mainly due to the longer alkyl chains of the IPAs filling in the void space at the bilayer center, allowing the alkyl chains to align more straightly along the bilayer normal with smaller tilt angles.

**Figure 6:**
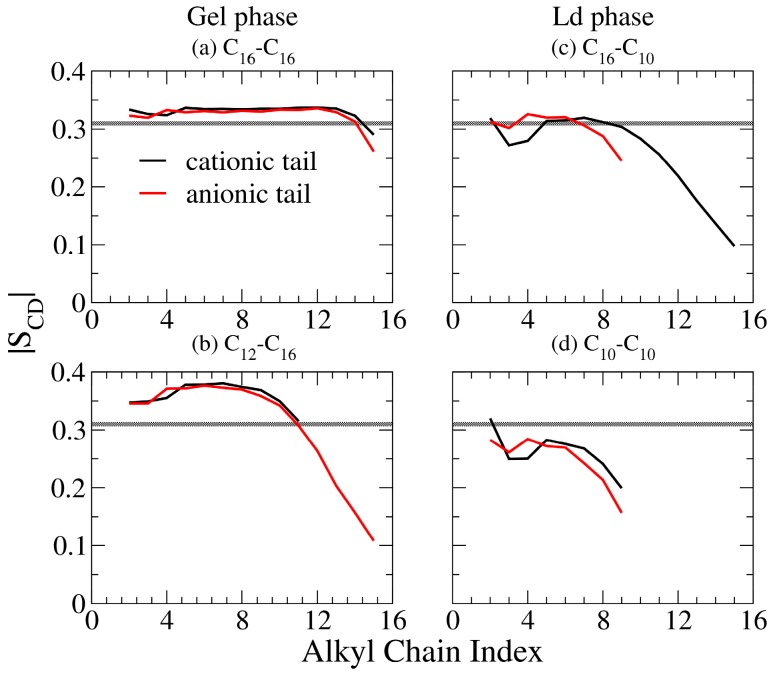
Representative deuterium order parameter |S_CD_| profiles for the cationic (black line) and the anionic (red line) components of the IPA systems (**a**) C_16_TMA^+^-C_16_S^−^ and (**b**) C_12_TMA^+^-C_16_S^−^ in gel (S) phase, and (**c**) C_16_TMA^+^-C_10_S^−^ and (**d**) C_10_TMA^+^-C_10_S^−^ systems in liquid-disordered (Ld) phase. The gray lines represent the threshold value of |S_CD_| = 0.31 to distinguish the Ld and S phases.

Deuterium order parameter (S_CD_) reflects the ordering of chain segment and gives one an information of the chain orientation toward the molecular tilting and chain ordering in different phases. From MD trajectories, S_CD_ can be evaluated as [35] follows:

(3) $$S_{\mathrm{CD}}=\frac{1}{2}\langle 3\cos^{2}\alpha -1\rangle$$

where α denotes the angle between the C-H bond vector and the bilayer normal. The plateau value of |S_CD_| is about 0.4 for a S phase phospholipid bilayer, and 0.2 for a Ld phase lipid bilayers [36,37]. The representative S_CD_ profiles for IPA bilayers in Figure 6 demonstrate that the alkyl chains within the Ld phase IPA bilayers are much disordered than that in the S phase, owing to the higher freedom of motion in the bilayer hydrophobic region. In addition, for an asymmetric IPA bilayer, the |S_CD_| value at the terminal segments decreases due to the alkyl chain mismatch. For complete |S_CD_| profiles for all tested IPA systems, please refer to Figures S6–S9 of Supplementary Materials.

**Figure 7:**
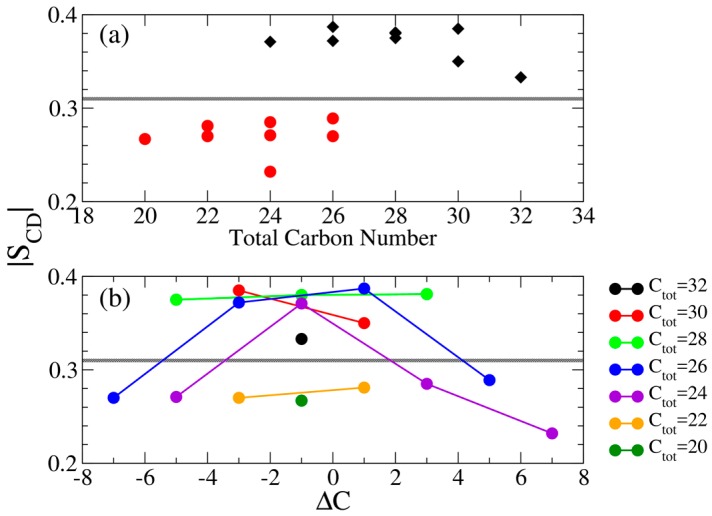
Averaged |S_CD_| value of middle segments plotted with respect to (**a**) the total alkyl carbon number (top panel) in which bilayers in S and Ld phases are colored in black diamonds and red dots, respectively, and (**b**) ΔC (bottom panel) in which each line represents systems with equal total alkyl carbon numbers. The gray lines represent the threshold value of |S_CD_| = 0.31 to distinguish the Ld and S phases.

From S_CD_ profiles, we observed flat plateau for IPA systems in S phase. Here, we defined the carbon atoms from 2nd alkyl carbon to the nth (or mth) carbon, if C_n_S^−^ (or C_m_TMA^+^) has the shorter alkyl chain, as the middle segments for the flat plateau. For instance, the middle segments of the C_14_TMA^+^-C_16_S^−^ IPA is defined from 2nd to 14th carbon atoms, and that of C_12_TMA^+^-C_16_S^−^ IPA is from 2nd to 12th carbons. The averaged |S_CD_| value of middle segments versus the total alkyl carbon atoms are shown in Figure 7. Generally, increasing the total alkyl chain length leads to a higher averaged |S_CD_| value for the Ld phase IPA bilayers, indicating an enhanced alkyl chain ordering induced by increased v.d.W interactions among longer chains. However, this trend is not as pronounced in the S phase system due to the variation of the alkyl chain tilt angle as shown in Figure 5. As listed in Table 2, the IPA systems in the S phase have averaged |S_CD_| values > 0.33, much different from that for IPA systems in the Ld phase valuing < 0.29. Therefore, based on the |S_CD_|, we defined a threshold |S_CD_| = 0.31 as the phase boundary to distinguish the S and Ld phases as shown in Table 2 and depicted in Figure 6 and Figure 7. 

**Figure 8:**
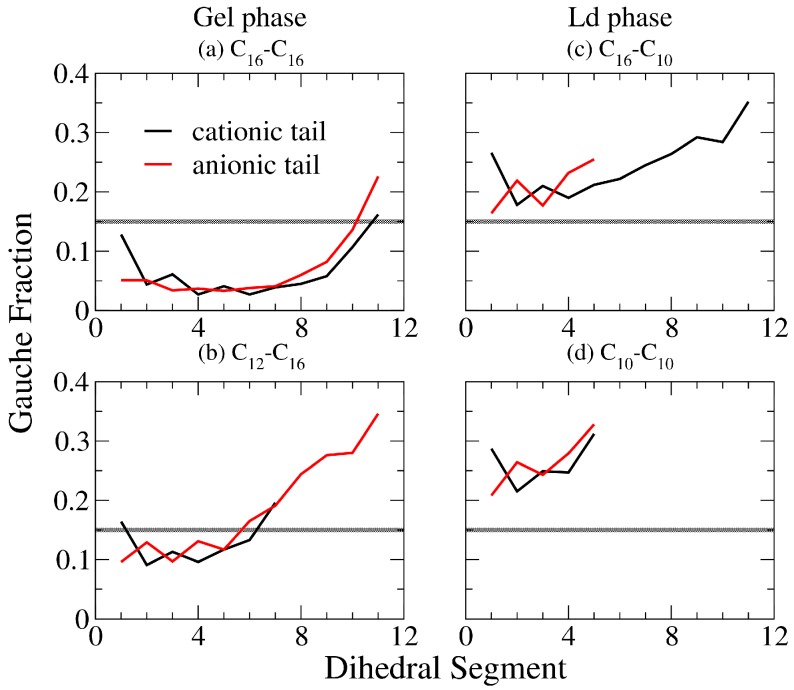
Representative gauche fraction profiles for the cationic (black line) and the anionic (red line) components of the IPA systems (**a**) C_16_TMA^+^-C_16_S^−^ and (**b**) C_12_TMA^+^-C_16_S^−^ in gel (S) phase, and (**c**) C_16_TMA^+^-C_10_S^−^ and (**d**) C_10_TMA^+^-C_10_S^−^ systems in liquid-disordered (Ld) phase. The gray lines represent the threshold gauche fraction value of 0.15 to distinguish the Ld and S phases.

In order to characterize the alkyl chain conformation within an IPA bilayer, we analyzed the gauche fraction for all the alkyl dihedral angles along an alkyl chain. Here, a dihedral segment was defined as a gauche conformer when its torsional angle was in the range of −120 to 120 degrees, and a trans conformer otherwise. Gauche fraction was then presented as the ratio of gauche conformation within the alkyl chain structure. The representative gauche fraction profiles for the C_12_TMA^+^-C_16_S^−^ IPA systems are shown in Figure 8. For S phase bilayers, the IPA molecules are well packed with higher alkyl chain ordering, resulting in gauche fraction values of below 0.15. In contrast, bilayers in the Ld phase have disordered alkyl chain packing, leading to gauche fraction values of greater than 0.15. Hence, a gauche value of 0.15 was defined as a boundary value for identifying the IPA phase condition as marked in Figure 8. Similar to the S_CD_ analysis, gauche fractions at the terminal segments increase as the |ΔC| increases, owing to the increased conformational freedom for the asymmetric alkyl chains. For complete gauche fraction profiles for all tested IPA systems, please refer to Figures S10–S13 of Supplementary Materials.

**Figure 9:**
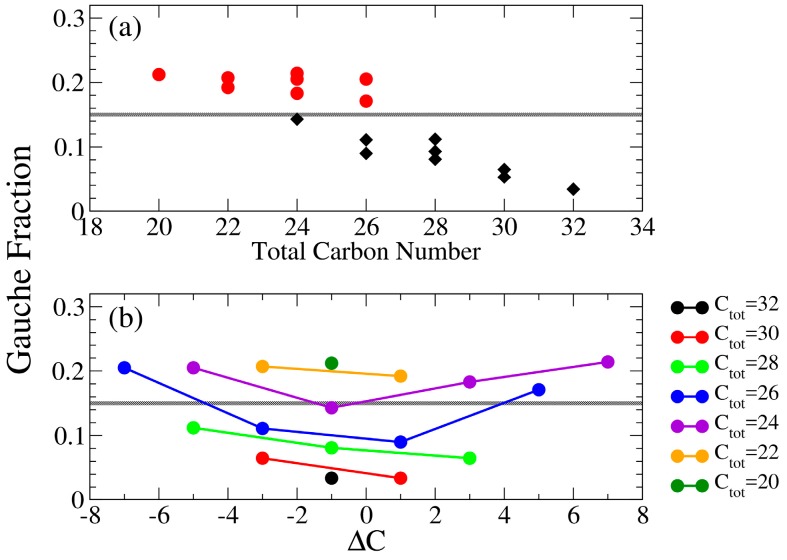
Averaged gauche fraction value of middle segments plotted with respect to (**a**) the total alkyl carbon number (top panel) in which bilayers in S and Ld phases are colored in black diamonds and red dots, respectively, and (**b**) ΔC (bottom panel) in which each line represents systems with equal total alkyl carbon numbers. The gray lines represent the threshold gauche fraction value of 0.15 to distinguish the Ld and S phases.

Both gauche fraction and S_CD_ analysis characterize the alkyl chain ordering. However, the calculation of S_CD_ involves taking reference with respect to the bilayer normal, and can be affected by both the alkyl chain tilting orientations and conformations. In contrast, the gauche fraction characterizes only the intra-molecular conformation of the alkyl chain segment. Figure 9 shows averaged gauche fraction values of the middle alkyl segments with respect to total alkyl chain length and ΔC for all the tested IPA systems. Compared with Figure 7, we found that both |S_CD_| and gauche fraction have distinct threshold values distinguishing the S and the Ld phases. Also, the averaged gauche fraction decreases as the total carbon number increases. This is due to the increased alkyl chain packing order induced by enhanced v.d.W interactions for elongated alkyl chain. Yet, due to the effect of tilting orientation, the |S_CD_| lacks of such obvious tendency as the gauche fraction profiles. Furthermore, the greater |ΔC| generally leads to lower value of averaged |S_CD_| and more gauche conformers, indicating a greater alkyl chain mismatch can reduce the alkyl chain ordering within the bilayer. 

### 3.4. IPA Bilayer Mechanical Properties

To examine the effects of total number of carbon atoms of alkyl chains and alkyl chain asymmetry on the bilayer mechanical properties, we evaluated the mechanical moduli for all the tested IPA bilayer structures either in the S or the Ld phase. We first analyzed the area expansion modulus, K_A_, to characterize the membrane resistance toward the lateral deformation. In MD simulations, K_A_ can be calculated from the lateral area fluctuation according to the linear response theory [38,39],

(4) $$k_{A}=\frac{k_{B}T\langle A\rangle }{N\langle \delta_{A}^{2}\rangle }$$

where $k_{B}$ is the Boltzmann constant, T is the simulation temperature of 298 K, N = 64 is the number of IPA per leaflet, and 〈A〉 and $\langle \delta_{A}^{2}\rangle$ denote the averaged area per molecule and the corresponding variance, respectively. 

**Figure 10:**
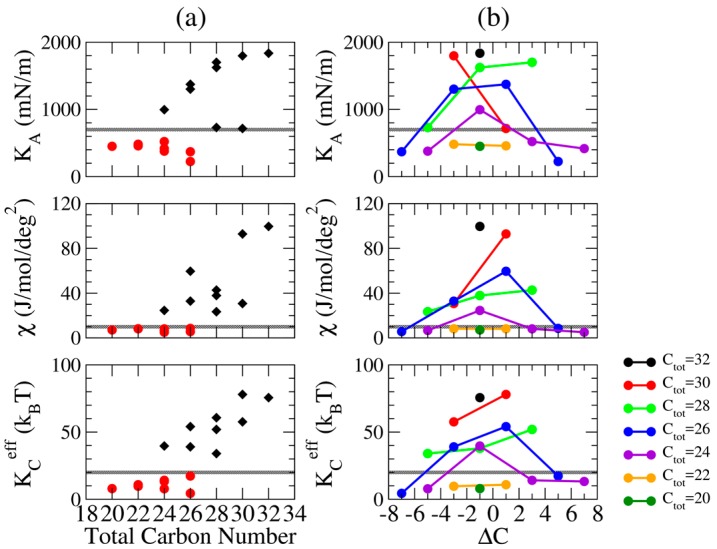
The area expansion modulus K_A_ (top panel), the molecular tilt modulus χ (second panel) and the effective bending rigidity $K_{C}^{eff}$ (bottom panel) for all the tested IPA systems with respect to (**a**) total number of carbon atoms where IPA bilayers in S and Ld phases at 298 K are colored in black diamonds and red dots, respectively, and (**b**) ΔC in which each line represents systems with equal total alkyl carbon numbers. The gray line represents the threshold values of K_A_ = 700 mN/m, χ = 10 J/mol/deg^2^, and $K_{C}^{eff}$ = 20 k_B_T to distinguish the Ld and S phases.

The resulting K_A_ for all the IPA systems are listed in Table 2. Compared with the phase boundary, we found a threshold K_A_ values of 700 mN/m that distinguishes the S and Ld phases. The K_A_ values for the S phase IPA systems are significantly higher than the ones for Ld phase systems. This is due to the solid-like feature for the well packed hydrophobic chains in the S phase bilayer, providing the membrane a greater resistance against the lateral deformation. In addition, as shown in Figure 10a, IPA bilayers with increased alkyl carbon atoms have greater K_A_. This is attributed to the increased v.d.W interaction within the hydrophobic region. Yet, as shown in Figure 10b, a greater |ΔC| leads to reduced K_A_ because of the decreased alkyl chain ordering induced by the alkyl chain mismatch.

Molecular tilt modulus χ characterizes the energy requirement for changing the alkyl chain tilting orientation. A higher molecular tilt modulus indicates each molecule within a IPA bilayer is prone to maintain their orientation against the external deformation. From MD trajectories, χ can be evaluated via the quadratic fitting of the tilting free energy profile F(θ), obtained via the Boltzmann’s inversion of the tilt angle distribution P(θ), e.g., Figure 5 [40].

(5) $$F\left(\theta \right)=-k_{B}Tln\left[\frac{P\left(\theta \right)}{\sin\theta }\right]=F\left(\theta_{0}\right)+\frac{\chi }{2}{\left(\theta -\theta_{0}\right)}^{2}$$

where *K_B_* and *T* are the Boltzmann constant and the simulation temperature of 298 K, $\theta_{0}$ denotes the equilibrium tilt angle, and sinθ is the Jacobian normalization factor, respectively. 

The resulting χ for all tested IPA system are listed in Table 2. Combined with the phase boundary, the S phase IPA bilayers have higher χ values than the Ld phase systems, and a boundary χ value of 10 J/mol/deg^2^ can be determined. The high χ arises from the well-packed IPA structure in the S phase, whereas the IPA alkyl chains in the Ld phase systems have more freedom to change their orientations. This result is also comparable with tilt angle distribution profiles in Figure 5, where Ld phase IPA bilayers have wider tilt angle distribution than the S phase system.

Figure 10a illustrates that, in the S phase, IPA bilayers with higher amount of total alkyl carbons have greater χ. However, in the Ld phase, IPA membranes have similar χ values. This suggests the increased v.d.W interaction for longer alkyl chains can enhance the tilting resistance of the alkyl tails against the external force. In contrast, the alkyl chain disordering can reduce the molecular tilt modulus. The combined effects leads to different responses of χ on the total alkyl chain length for the S and Ld phase membranes. Moreover, as shown in Figure 10b, the IPA systems with higher |ΔC| are generally exhibits greater χ values, mainly due to the loose packing near the IPA bilayer hydrophobic core region induced by the alkyl chain mismatch.

The effective bending rigidity $K_{C}^{eff}$ for an IPA bilayer characterizes the membrane resistance toward the bending deformation. Here, $K_{C}^{eff}$ was estimated from the weighting sum the splay modulus ($\chi_{ij}$) [17] as follows:

(6) $$\frac{1}{K_{C}^{eff}}=\left(\frac{1}{\sum \varphi_{ij}}\right)\sum \frac{\varphi_{ij}}{\chi_{ij}}\;,$$

where $\varphi_{ij}$ denotes the number of molecule *i*-*j* pairs. The molecular splay modulus $\chi_{ij}$ characterizes the free energy cost for splaying the *i*- and *j*-type molecules in the membrane. Similar to the molecular tilt modulus χ in Equation (5), $\chi_{ij}$ can be evaluated from the quadratic fitting of the splay angle free energy profile obtained from the Boltzmann inversion of the splay angle distribution [17,22]. Larger value of $K_{C}^{eff}$ indicates the bilayer has higher resistance toward the bending deformation, which corresponds to a greater energy cost for splaying two molecules within the bilayer. 

$K_{C}^{eff}$ values for all the tested IPA systems are listed in Table 2. Similar to the other mechanical moduli, a threshold $K_{C}^{eff}$ values of 20 k_B_T were defined as the boundary value for the S and the Ld phase systems. In the S phase, the solid-like characters for the well packed IPA structures lead to higher $K_{C}^{eff}$ value compared with the ones for the Ld phase membranes. Figure 10 further illustrates the effects of total alkyl chain length and the alkyl chain asymmetry on the effective bending rigidity. IPA systems in the S phase exhibit larger $K_{C}^{eff}$ with the longer total alkyl chain length, suggesting the increased v.d.W interaction improves the bilayer effective bending rigidity. In addition, IPA bilayers of greater |ΔC| values have smaller $K_{C}^{eff}$ for IPA bilayers in both the S and the Ld phases. This validates the influences of alkyl carbon atom mismatch on the overall bending rigidity.

Combining the presented structural and mechanical analyses, we found that increasing the total alkyl chain length can enhance the v.d.W interaction within the IPA bilayer hydrophobic region, leading to higher alkyl chain ordering and improved mechanical strengths. In contrast, increasing the alkyl chain asymmetry of IPA, i.e., increased |ΔC|, reduces the chain ordering within the bilayer hydrophobic region and the overall mechanical moduli of the membrane. Elongating the C_m_TMA^+^-C_n_S^−^ IPA chain to m, n ≥ 12 can further lead to the Ld to S phase transition for the bilayer at 298 K, where the S phase exhibit a tight alkyl chain packing and much greater mechanical moduli. 

## 4. Conclusions

The molecular dynamics (MD) simulation was exploited to examine the bilayer structural and mechanical properties for the IPA system of alkyltrimethyl-ammonium–alkylsulfate series, i.e., C_m_TMA^+^-C_n_S^−^. With various combinations of m and n ranging from 10 to 16, we evaluated the effects of the total alkyl carbon number and the alkyl chain asymmetry of IPAs on the structural and mechanical properties of the corresponding bilayers. The tested IPA bilayer were found to exhibit in either the Ld phase or the gel phase at 298 K depending on the total alkyl carbon number and the IPA complex asymmetry. As summarized in Table 2, further increasing the alkyl chain length of C_m_TMA^+^-C_n_S^−^ IPA to m and n ≥ 12 leads to a transition from Ld to S phase for the biomimetic bilayer at 298 K. To distinguish the Ld and S phases, we defined the threshold values of A_IPA_ = 0.45 nm^2^, |S_CD_| = 0.31, and alkyl gauche fraction of 0.15 for membrane structural properties and K_A_ = 700 mN/m, χ = 10 J/mol/deg^2^, and $K_{C}^{eff}$ = 20 k_B_T for bilayer mechanical properties.

Increasing the total alkyl chain length of IPA leads to enhanced v.d.W. interactions within the hydrophobic region of the bilayer and therefore improves the alkyl chain packing order and the overall mechanical moduli. According to the density profiles, the nitrogen and sulfur atoms of the cationic and anionic components are aligned on the same transverse position along the IPA bilayer normal. Compared with the IPA molecular structures, we therefore defined the asymmetry index, ΔC = m − (n + 1), to characterize the alkyl chain mismatch for the C_m_TMA^+^-C_n_S^−^ IPAs. A greater |ΔC| value of IPA, i.e., more asymmetric alkyl chain combination, results in less alkyl chain ordering, and the reduced overall mechanical moduli for the bilayer system. Note that all the tested IPA are asymmetric, according to the asymmetry index ΔC. To generate a symmetric IPA complex, one may need to construct the alkyltrimethylamine–alkylsulfate IPA with odd alkyl carbon number on either one of the alkyl chains, or choosing other amphiphiles with different charged head groups, such as alkylsulfonate. The combined results provide detailed structural and mechanical characteristics at molecular level for biomimetic IPA bilayers, which is helpful for designing IPA membrane systems for various applications. 

## Figures and Tables

**Table 1:** Combination of alkyltrimethylammonium–alkylsulfate IPA systems, i.e., C_m_TMA^+^-C_n_S^−^, with m and n valued from 16 to 10. The surfactant abbreviations, the total alkyl carbon number, and the asymmetry index ΔC (Equation (1)) are also given.

m-n	16	14	12	10	
16	16-16	16-14	16-12	16-10	m-n
	HTMA-HS	HTMA-TS	HTMA-DS	HTMA-DeS	Abbreviation
	32	30	28	26	Total Carbon
	−1	1	3	5	ΔC
14	14-16	14-14	14-12	14-10	
	TTMA-HS	TTMA-TS	TTMA-DS	TTMA-DeS	
	30	28	26	24	
	−3	−1	1	3	
12	12-16	12-14	12-12	12-10	
	DTMA-HS	DTMA-TS	DTMA-DS	DTMA-DeS	
	28	26	24	22	
	−5	−3	−1	1	
10	10-16	10-14	10-12	10-10	
	DeTA-HS	DeTA-TS	DeTA-DS	DeTA-DeS	
	26	24	22	20	
	−7	−5	−3	−1	

**Table 2:** Structural and mechanical characteristics for all the tested C_m_TMA^+^-C_n_S^−^ IPA systems. The m-n combination, area per IPA complex A_IPA_ (nm^2^), area expansion modulus K_A_ (mN/m), molecular tilt modulus χ (J/mol/deg^2^), effective bending rigidity $K_{C}^{eff}$ (k_B_T), average |S_CD_| and gauche factions for IPA middle alkyl segments, and experimental transition temperature (T_m_) are noted sequentially for each system [12]. Red line shows the phase-boundary dividing the S and the Ld phases: IPA systems with both m ≥ 12 and n ≥ 12 are in the gel phase, and otherwise (m or n ≤ 10) in the Ld phase. The threshold values of membrane properties corresponding to the phase boundary are also listed.

m-n	16	14	12	10	
**16**	16-16	16-14	16-12	16-10	m-n
	0.425	0.428	0.428	0.476	A_IPA_ (nm^2^)
	1835.75	716.70	1701.20	225.76	K_A_ (mN/m)
	99.56	92.85	42.72	8.55	χ (J/mol/deg^2^)
	75.62	78.00	51.93	17.29	$K_{C}^{eff}$ (k_B_T)
	0.333	0.305	0.381	0.289	|S_CD_|
	0.034	0.053	0.093	0.171	Gauche Fraction
	338.28	339.83	327.09	N/A	T_m_
**14**	14-16	14-14	14-12	14-10	
	0.420	0.427	0.420	0.486	
	1797.40	1623.82	1373.57	521.80	
	30.72	37.86	59.55	8.14	
	57.59	60.71	54.05	14.12	
	0.385	0.380	0.387	0.285	
	0.065	0.081	0.090	0.183	
	N/A	328.28	332.80	N/A	
**12**	12-16	12-14	12-12	12-10	*Threshold Values:*
	0.430	0.431	0.440	0.492	A_IPA_ = 0.45 nm^2^
	730.84	1301.73	995.82	457.74	K_A_ = 700 mN/m
	23.45	32.85	24.53	8.18	χ = 10 J/mol/deg^2^
	34.02	39.03	39.67	10.85	$K_{C}^{eff}$ = 20 k_B_T
	0.375	0.372	0.371	0.281	|S_CD_| = 0.31
	0.112	0.111	0.143	0.192	X_Gauche_ = 0.15
	N/A	320.05	314.52	N/A	
**10**	10-16	10-14	10-12	10-10	
	0.495	0.499	0.499	0.500	
	370.90	379.41	481.92	451.00	
	5.68	6.71	8.24	7.14	
	4.40	7.83	9.67	7.94	
	0.270	0.271	0.270	0.267	
	0.205	0.205	0.207	0.212	
	N/A	303.71	304.06	N/A	

## Supplementary Materials

The following are available online at www.mdpi.com/2306-5354/4/4/84/s1, Tables S1 and S2: molecular topologies for IPA, Figures S1 and S2: equilibration verifications. Figures S3–S5: transverse density profiles, Figures S6–S9: deuterium order parameter |S_CD_| profiles, and Figures S10–S13: gauche fraction profiles for all the tested IPA bilayer systems.
