# Supplementary material for: The Effects of Alkyl Chain Combinations on the Structural and Mechanical Properties of Biomimetic Ion Pair Amphiphile Bilayers
Source: Bioengineering (Basel). 2017 Oct 11;4(4):84. doi: 10.3390/bioengineering4040084 (PMC5746751; doi:10.3390/bioengineering4040084)
Supplement: Supplementary file 1 [file bioengineering-04-00084-s001.pdf]

Table S1. Each atom corresponds to the atom type and the charges for the cationic surfactant of IPA systems, i.e. $C_{16}TMA^+$ (HTMA).

Atom	Atom type	charge
N	NTL	-0.600
C1	CTL2	-0.100
C2	CTL5	-0.350
C3	CTL5	-0.350
C4	CTL5	-0.350
H11	HL	0.250
H12	HL	0.250
H21	HL	0.250
H22	HL	0.250
H23	HL	0.250
H31	HL	0.250
H32	HL	0.250
H33	HL	0.250
H41	HL	0.250
H42	HL	0.250
H43	HL	0.250
C5	CTL2	-0.180
H51	HAL2	0.090
H52	HAL2	0.090
C6	CH2E	0.000
C7	CH2E	0.000
C8	CH2E	0.000
C9	CH2E	0.000
C10	CH2E	0.000
C11	CH2E	0.000
C12	CH2E	0.000
C13	CH2E	0.000
C14	CH2E	0.000
C15	CH2E	0.000
C16	CH2E	0.000
C17	CH2E	0.000
C18	CH2E	0.000
C19	CH2E	0.000

```
            H32
             |
        H31-C3-H33
             |
      H23    |   H43
       |     |    |
   H22-C2---N---C4-H42
       |     |    |
      H21    |   H41
             |
             |
             |
        H11-C1-H12
             |
             |
             |
             |
             |
             |
      H51---C5---H52
             |
             |
             C6
             C7
             C8
             C9
            C10
            C11
            C12
            C13
            C14
            C15
            C16
            C17
            C18
            C19
```

Table S2. Each atom corresponds to the atom type and the charges for the anionic surfactant of IPA systems, i.e. $C_{16}S^-$ (HS).

Atom	Atom type	charge
S	SL	1.333
OS1	OSL	-0.280
OS2	O2L	-0.650
OS3	O2L	-0.650
OS4	O2L	-0.640
C1	CTL2	-0.280
H11	HAL2	0.090
H12	HAL2	0.090
C2	CTL2	-0.180
H21	HAL2	0.090
H22	HAL2	0.090
C3	CH2E	0.000
C4	CH2E	0.000
C5	CH2E	0.000
C6	CH2E	0.000
C7	CH2E	0.000
C8	CH2E	0.000
C9	CH2E	0.000
C10	CH2E	0.000
C11	CH2E	0.000
C12	CH2E	0.000
C13	CH2E	0.000
C14	CH2E	0.000
C15	CH2E	0.000
C16	CH2E	0.000

```
     OS3
      |
  OS2-S-OS4
      |
     OS1
      |
 H11-C1-H12
      |
      |
 H21-C2-H22
      |
      |
      C3
      C4
      C5
      C6
      C7
      C8
      C9
     C10
     C11
     C12
     C13
     C14
     C15
     C16
```

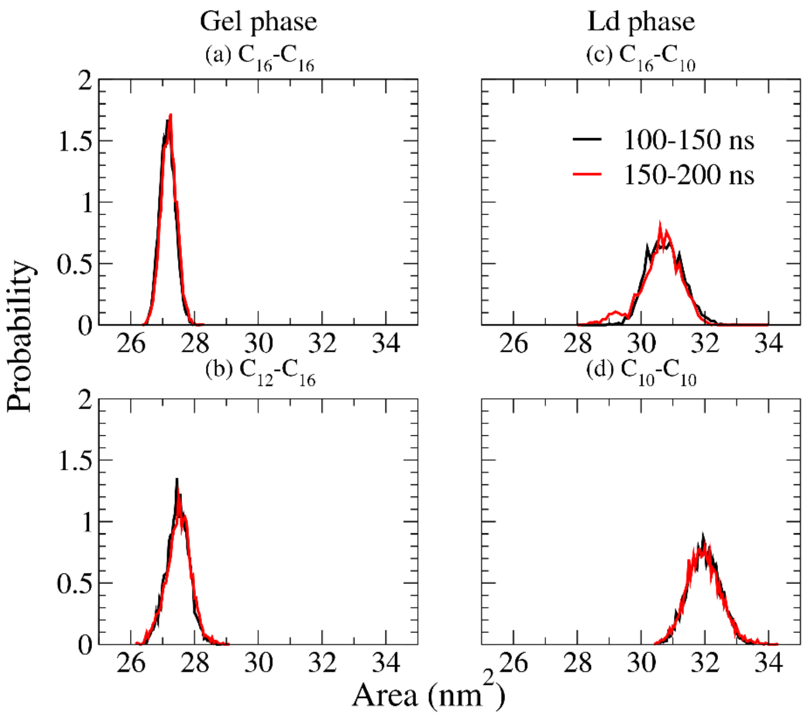

Fig. S1 The area distributions for the IPA systems of C_{16}-C_{16}, C_{16}-C_{10}, C_{12}-C_{16}, C_{10}-C_{10} from the first 50 ns (black line) and the second 50 ns MD simulations (red line).

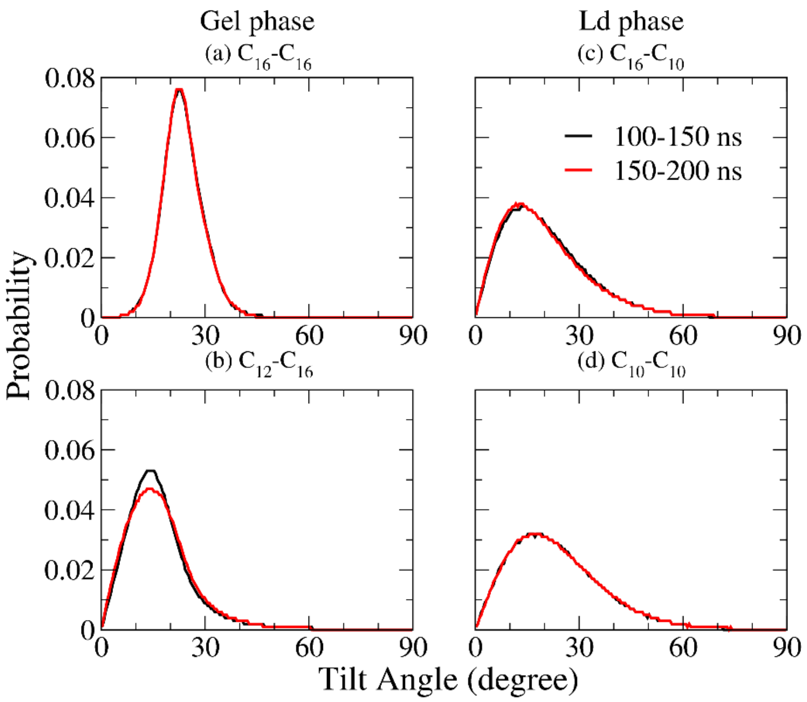

Fig. S2 The tilt angle distributions for the IPA systems of C_{16}-C_{16}, C_{16}-C_{10}, C_{12}-C_{16}, C_{10}-C_{10} from the first 50 ns (black line) and the second 50 ns MD simulations (red line).

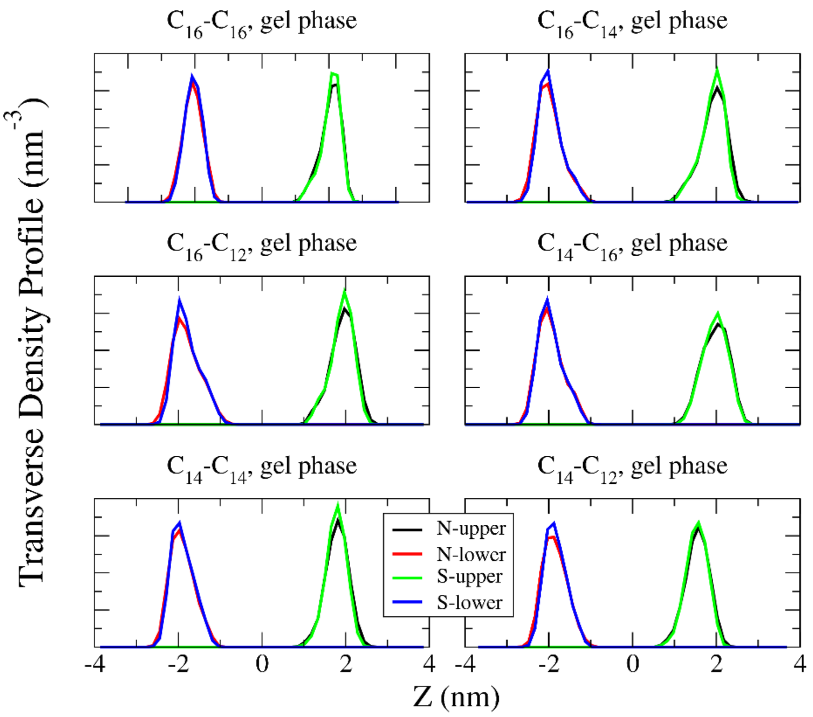

Fig. S3 Transverse density profiles of nitrogen and sulfur of the charged head groups for the IPA systems of C_{16}-C_{16}, C_{16}-C_{14}, C_{16}-C_{12}, C_{14}-C_{16}, C_{14}-C_{14}, C_{14}-C_{12}, which are all in the gel phase

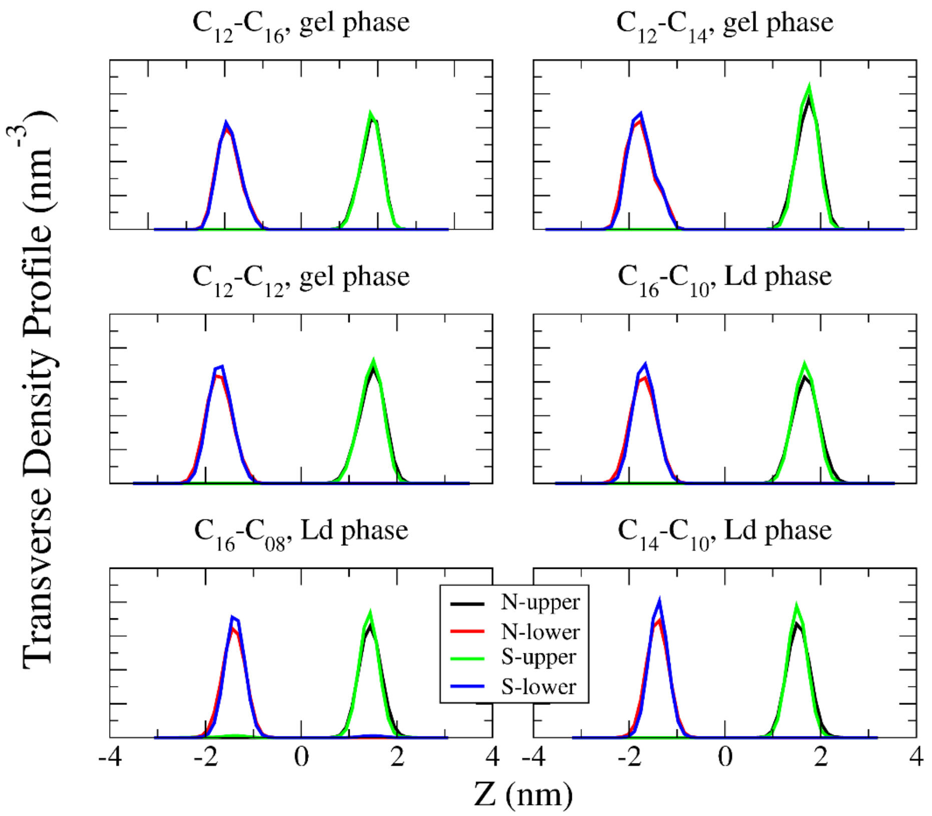

Fig. S4 Transverse density profiles of nitrogen and sulfur of the charged head groups for the IPA systems of C_{12}-C_{16}, C_{12}-C_{14}, C_{12}-C_{12}, C_{16}-C_{10}, C_{16}-C_{08}, C_{14}-C_{10}. The phase for each system is also given.

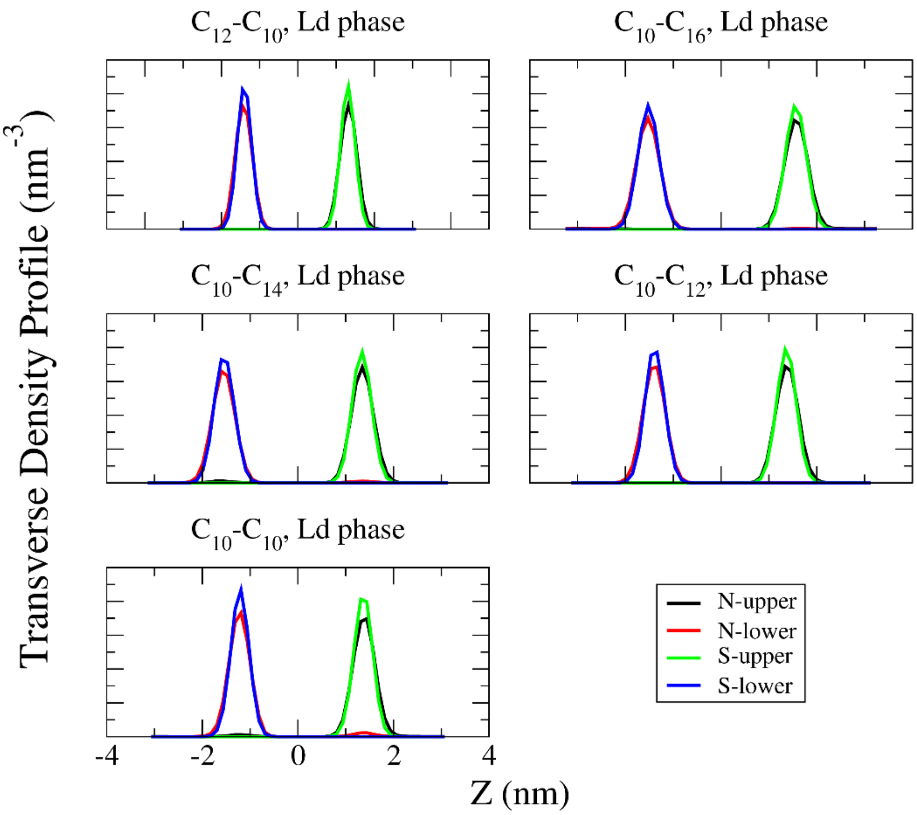

Fig. S5 Transverse density profiles of nitrogen and sulfur of the charged head groups for the IPA systems of C_{12}-C_{10}, C_{10}-C_{16}, C_{10}-C_{14}, C_{10}-C_{12}, C_{10}-C_{10}, which are all in the Ld phase.

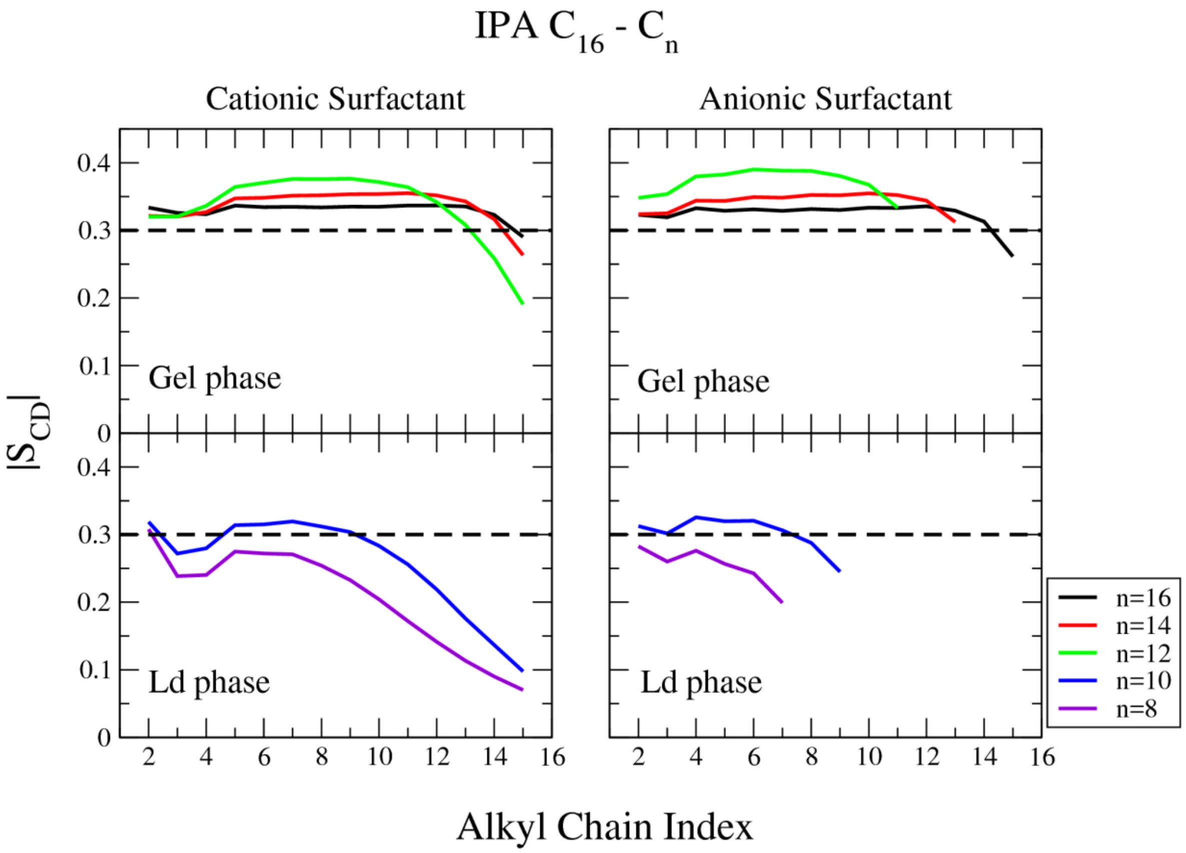

Fig. S6 Deuterium order parameter, S_{CD}, profiles for C_mTMA^+-C_nS^-, m = 16 IPA combinations, *i.e.* C_{16}-C_{16}, C_{16}-C_{14}, C_{16}-C_{12}, C_{16}-C_{10} and C_{16}-C_8 systems. Left and right columns are the S_{CD} profiles for the cationic and anionic components, respectively, for the IPA system in the gel phase (top) or the Ld phase (bottom).The dash line represents the threshold S_{CD} values of 0.3 which roughly distinguish the gel and Ld phase.

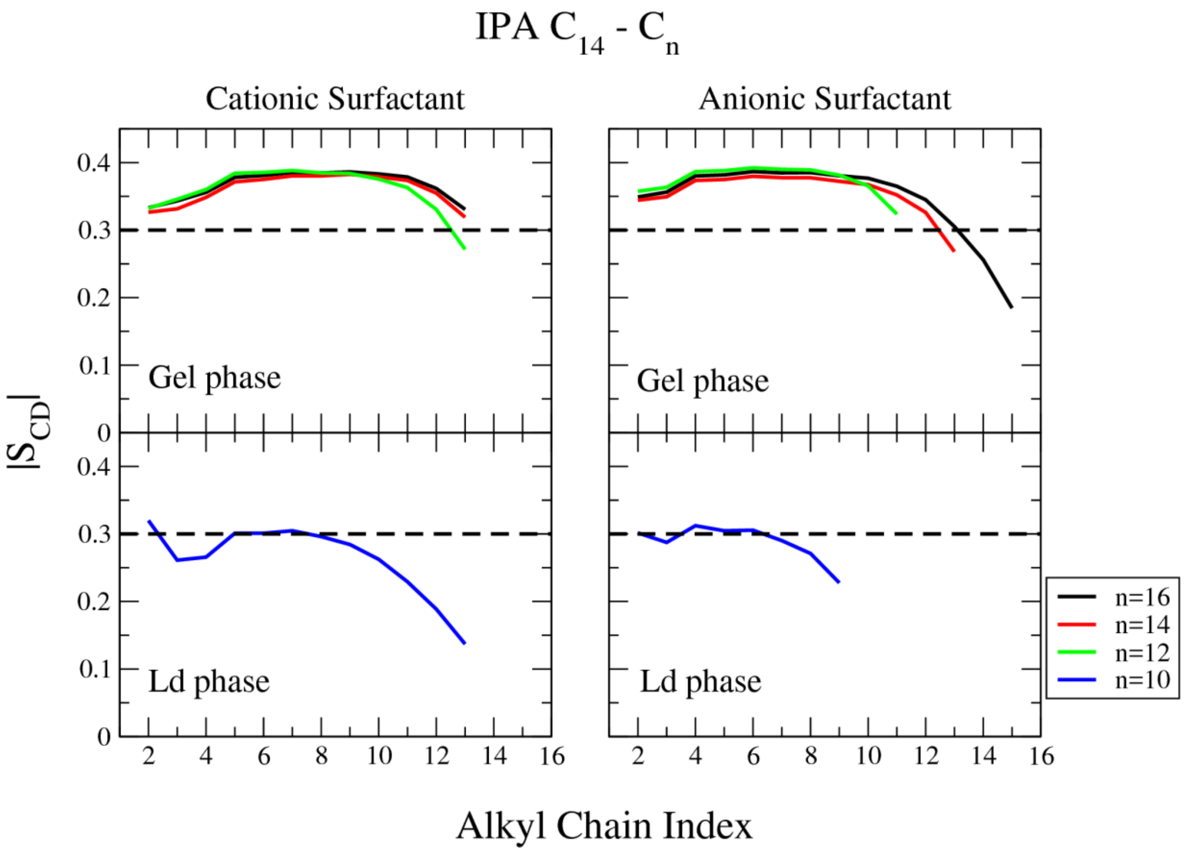

Fig. S7 Deuterium order parameter, S_{CD}, profiles for C_mTMA^+-C_nS^-, m = 14 IPA combinations, *i.e.* C_{14}-C_{16}, C_{14}-C_{14}, C_{14}-C_{12}, and C_{14}-C_{10} systems. Left and right columns are the S_{CD} profiles for the cationic and anionic components, respectively, for the IPA system in the gel phase (top) or the Ld phase (bottom). The dash line represents the threshold S_{CD} values of 0.3 which roughly distinguish the gel and Ld phase.

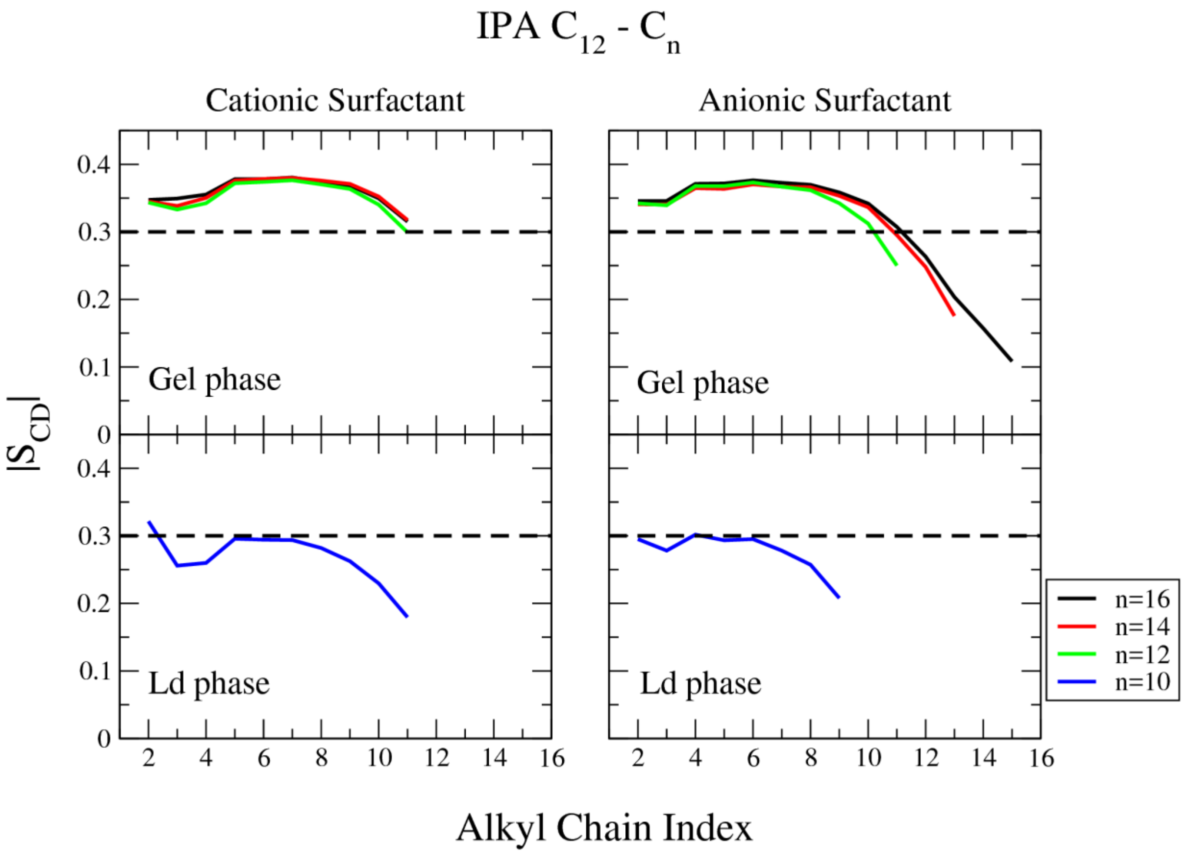

Fig. S8 Deuterium order parameter, S_{CD}, profiles for C_mTMA^+-C_nS^-, m = 12 IPA combinations, *i.e.* C_{12}-C_{16}, C_{12}-C_{14}, C_{12}-C_{12}, and C_{12}-C_{10} systems. Left and right columns are the S_{CD} profiles for the cationic and anionic components, respectively, for the IPA system in the gel phase (top) or the Ld phase (bottom). The dash line represents the threshold S_{CD} values of 0.3 which roughly distinguish the gel and Ld phase.

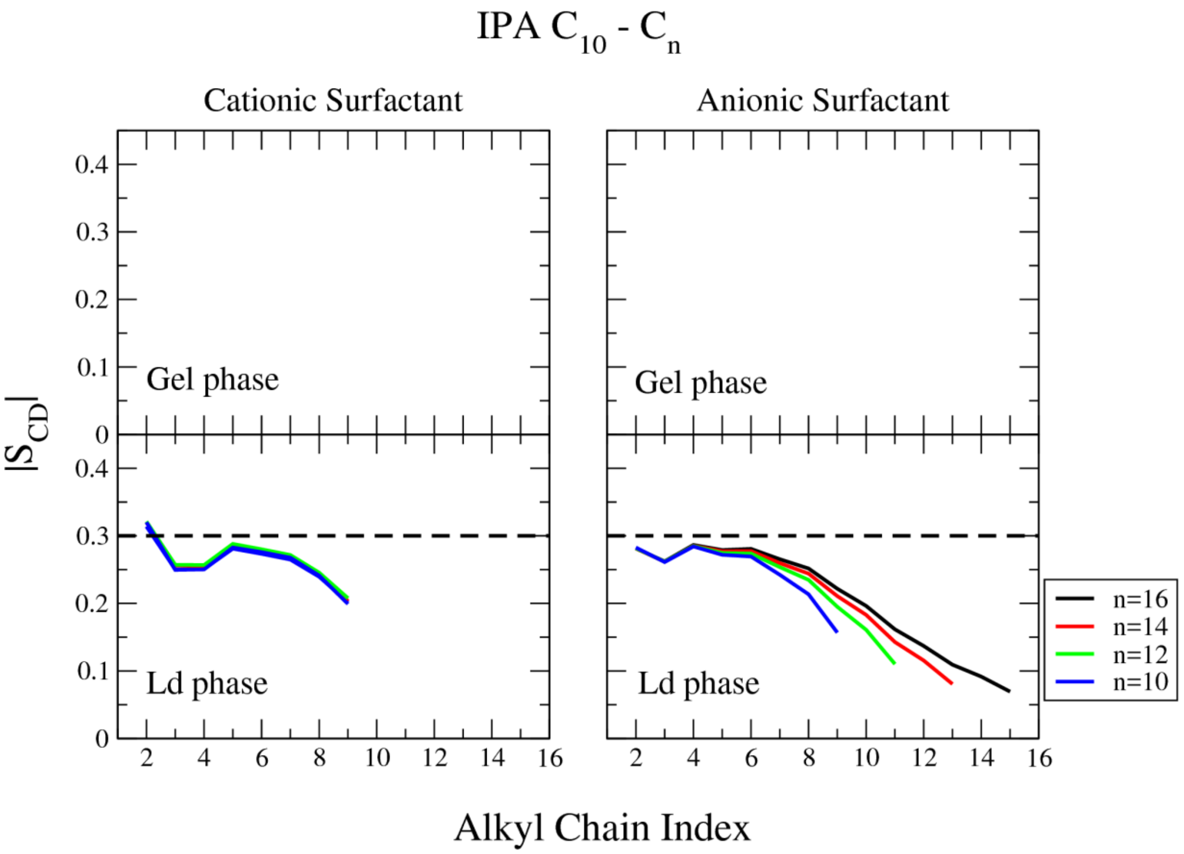

Fig. S9 Deuterium order parameter, S_{CD}, profiles for C_mTMA^+-C_nS^-, m = 10 IPA combinations, *i.e.* C_{10}-C_{16}, C_{10}-C_{14}, C_{10}-C_{12}, and C_{10}-C_{10} systems. Left and right columns are the S_{CD} profiles for the cationic and anionic components, respectively, for the IPA system in the gel phase (top) or the Ld phase (bottom). The dash line represents the threshold S_{CD} values of 0.3 which roughly distinguish the gel and Ld phase. With the m and n combination where m=10, all IPA systems are in the Ld phase.

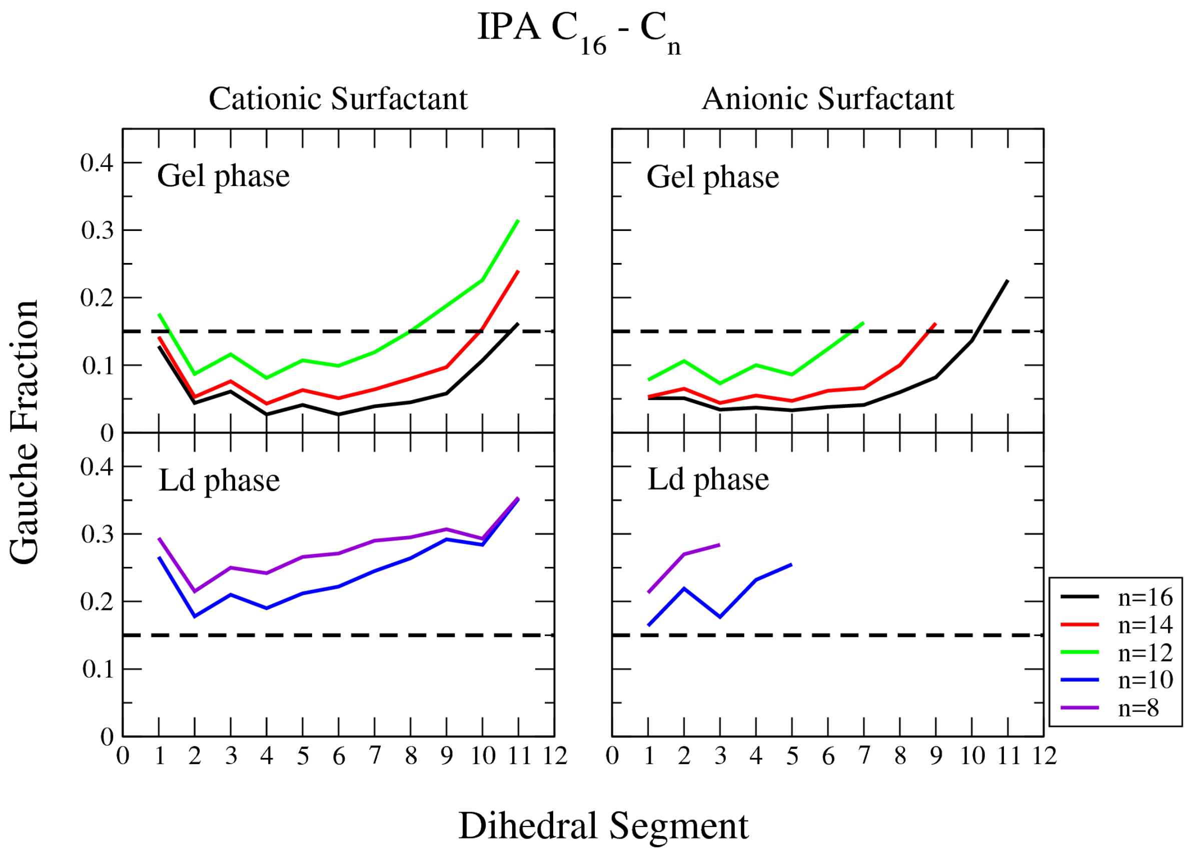

Fig. S10 Gauche fraction profiles for C_mTMA^+-C_nS^-, m = 16 IPA combinations, *i.e.* C_{16}-C_{16}, C_{16}-C_{14}, C_{16}-C_{12}, C_{16}-C_{10} and C_{16}-C_8 systems. Left and right columns are the gauche fraction profiles for the cationic and anionic components, respectively, for the IPA system in the gel phase (top) or the Ld phase (bottom). The dash line represents the threshold gauche fraction values of 0.15 which roughly distinguish the gel and Ld phase.

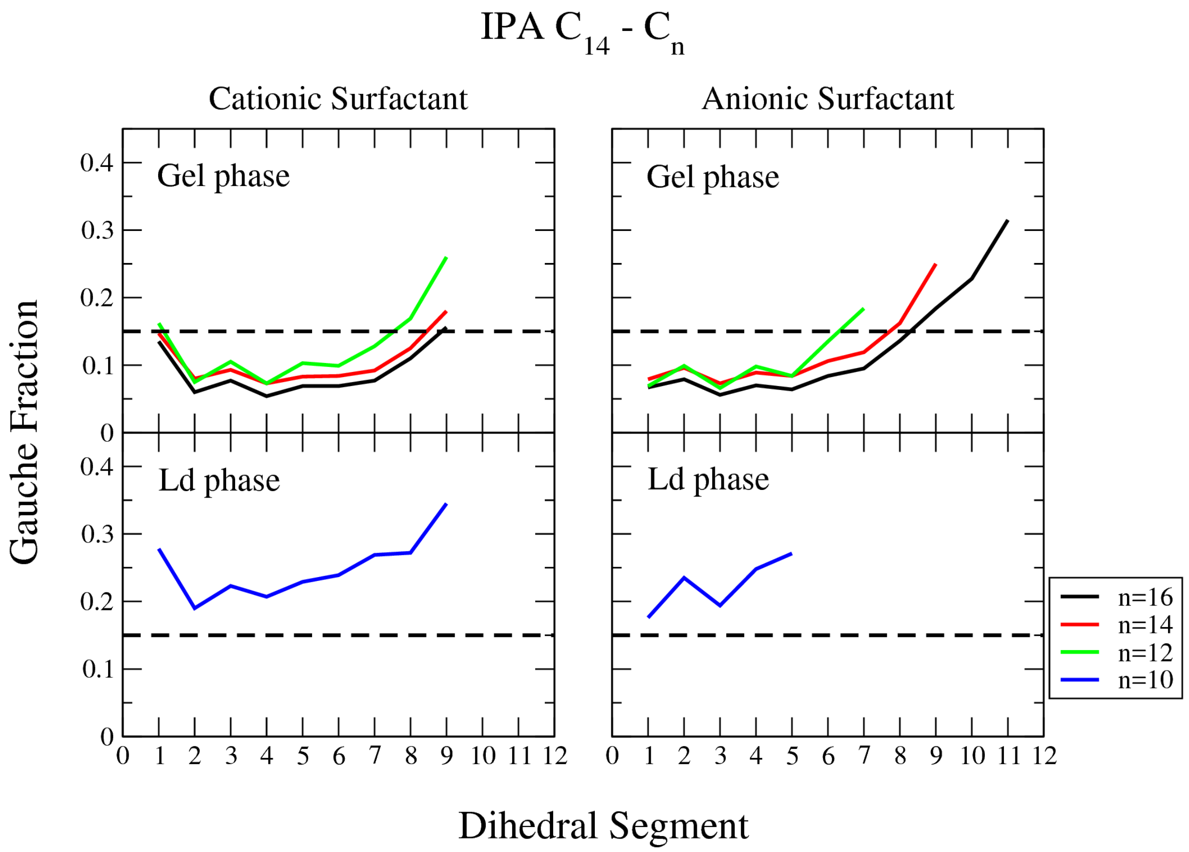

Fig. S11 Gauche fraction profiles for C_mTMA^+-C_nS^-, m = 14 IPA combinations, *i.e.* C_{14}-C_{16}, C_{14}-C_{14}, C_{14}-C_{12}, and C_{14}-C_{10} systems. Left and right columns are the gauche fraction profiles for the cationic and anionic components, respectively, for the IPA system in the gel phase (top) or the Ld phase (bottom). The dash line represents the threshold gauche fraction values of 0.15 which roughly distinguish the gel and Ld phase.

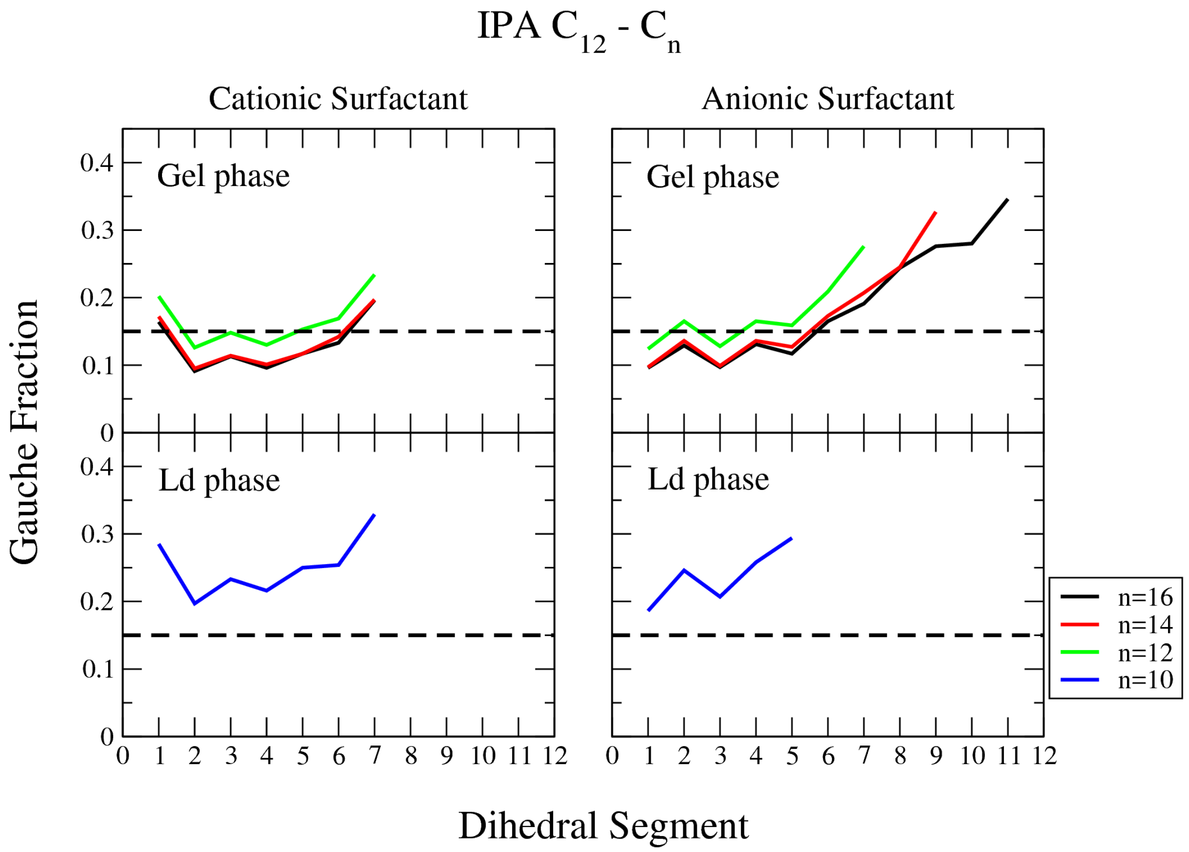

Fig. S12 Gauche fraction profiles for C_mTMA^+-C_nS^-, m = 12 IPA combinations, *i.e.* C_{12}-C_{16}, C_{12}-C_{14}, C_{12}-C_{12}, and C_{12}-C_{10} systems. Left and right columns are the gauche fraction profiles for the cationic and anionic components, respectively, for the IPA system in the gel phase (top) or the Ld phase (bottom). The dash line represents the threshold gauche fraction values of 0.15 which roughly distinguish the gel and Ld phase.

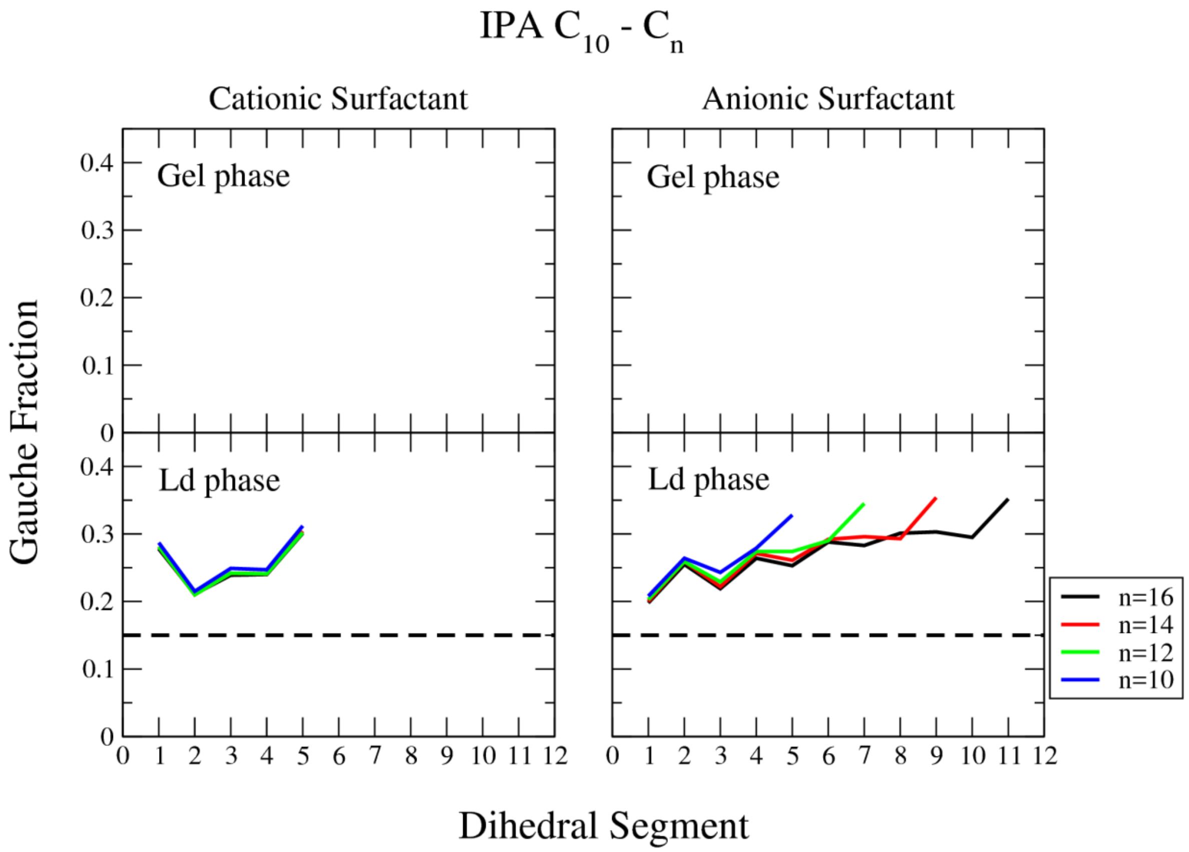

Fig. S13 Gauche fraction profiles for C_mTMA^+-C_nS^-, m = 10 IPA combinations, *i.e.* C_{10}-C_{16}, C_{10}-C_{14}, C_{10}-C_{12}, and C_{10}-C_{10} systems. Left and right columns are the gauche fraction profiles for the cationic and anionic components, respectively, for the IPA system in the gel phase (top) or the Ld phase (bottom). The dash line represents the threshold gauche fraction values of 0.15 which roughly distinguish the gel and Ld phase. With the m and n combination where m=10, all IPA systems are in the Ld phase.
